# Functional dissimilarity in mixed forests promotes stem radial growth by mitigating tree water deficit

**DOI:** 10.1093/nsr/nwad320

**Published:** 2023-12-18

**Authors:** Hong-Tu Zhang, Gheyur Gheyret, Yun-Hao Bai, Yanpei Guo, Shan Li, Bernhard Schmid, Helge Bruelheide, Keping Ma, Zhiyao Tang

**Affiliations:** Institute of Ecology, College of Urban and Environmental Sciences and Key Laboratory for Earth Surface Processes of Ministry of Education, Peking University, China; Institute of Ecology, College of Urban and Environmental Sciences and Key Laboratory for Earth Surface Processes of Ministry of Education, Peking University, China; College of Geography and Tourism, Xinjiang Normal University, China; Institute of Ecology, College of Urban and Environmental Sciences and Key Laboratory for Earth Surface Processes of Ministry of Education, Peking University, China; Institute of Ecology, College of Urban and Environmental Sciences and Key Laboratory for Earth Surface Processes of Ministry of Education, Peking University, China; State Key Laboratory of Vegetation and Environmental Change, Institute of Botany, Chinese Academy of Sciences, China; Institute of Ecology, College of Urban and Environmental Sciences and Key Laboratory for Earth Surface Processes of Ministry of Education, Peking University, China; Department of Geography, Remote Sensing Laboratories, University of Zurich, Switzerland; Institute of Biology/Geobotany and Botanical Garden, Martin Luther University Halle-Wittenberg, Germany; German Centre for Integrative Biodiversity Research (iDiv) Halle-Jena-Leipzig, Germany; State Key Laboratory of Vegetation and Environmental Change, Institute of Botany, Chinese Academy of Sciences, China; Institute of Ecology, College of Urban and Environmental Sciences and Key Laboratory for Earth Surface Processes of Ministry of Education, Peking University, China

Tree growth is a key component of forest ecosystem functioning and is well-known to be limited by water availability [1]. It is acknowledged that composition and diversity of co-occurring tree species can mitigate the influence of drought on tree growth through the resource complementarity for water use via niche partitioning and facilitation between different species [2,3]. However, direct evidence on the role of water use in diversity-production relationships remains limited.

For individual trees, species interactions at the neighbourhood scale play a major role in water supply and growth [4], which provide insights into the emerging net effects of biodiversity at the community scale. Such effects might be achieved by differences in species’ functional traits. These differences in functional trait composition can be quantified at the neighbourhood scale in several ways to provide mechanistic insights into the processes underlying diversity effects. The community-weighted mean (CWM) of trait values emphasizes the dominant trait values in a community. In this sense, CWM of a trait associated with water capture may reflect the competitive intensity for water, and more water-acquisitive species at the neighbourhood scale can reduce the amount of water available to focal individuals. In contrast, the functional diversity (FD) of a community reflects the variation of resource use strategies among co-occurring species and can serve as a proxy for niche differentiation within neighbourhoods [5].

Neighbourhood complementarity is also modulated by the identity of the focal tree, which is inevitably ignored at the community scale [4]. Individual characteristics, such as tree size and resource-capture strategy, determine how trees perform and interact with their neighbours [6]. For example, the water use of an individual tree is influenced by its size, such that competition for water is likely size-asymmetric [6]. In addition, the effect of neighbourhood composition on individuals’ drought resistance can be mediated by the drought-tolerant traits of species [2]. This indicates that it is possible that individual-mediated neighbourhood interactions can shape the water status of focal individuals.

In this study, we explored the effects of neighbourhood on tree water deficit (TWD) and the relationships between TWD and stem radial growth of individuals. TWD extracted from dendrometer data quantifies the loss of water from elastic tissues and is a proxy for reflecting drought stress and water status of trees [7]. To do so, we used high-frequency (every 30 min) dendrometer measurements taken from 160 trees of 12 focal species in a large-scale tree diversity manipulation experiment platform in subtropical China, Biodiversity-Ecosystem Functioning Experiment (BEF)-China platform [8]. These trees were planted in plots and experimentally maintained with species richness treatments of 1, 2, 4, 8, 16, or 24 species in Dexing, Jiangxi Province, where subtropical forests experience more physiological drought in warm and dry environments under climate change [9]. We considered multiple functional traits including life habit, xylem hydraulic traits, stomata traits as well as leaf economics spectrum that play a role in resource acquisition of water, carbon and nutrients [10]. We examined how the CWM and FD of these functional traits at the neighbourhood scale influence TWD of the focal tree. We hypothesized that neighbourhood effects depend on both the size and functional trait value of the focal trees (H1). Furthermore, we hypothesized that neighbouring trees can influence stem radial growth by water resource complementarity, that reduced TWD can increase stem radial growth (H2).

Of the 12 focal species (Table S1), we found divergent annual TWD and radial growth among species and between evergreen and deciduous trees (Figs S3 and S4). TWD exhibited considerable seasonal variation, with the lowest values typically in summer and higher values in spring and winter, corresponding to the changes of precipitation and soil moisture (Fig. S5). TWD is a strong and instantaneous response to declines in atmospheric and soil moisture, which is more prolonged and severe in dry seasons [7]. Among the 32 species-level traits, we selected xylem pressure sensitivity (S) in the final analysis that explained the most variance in TWD (Table S2). Xylem pressure sensitivity is measured as the inverse slope of the xylem vulnerability curve, depicting the sensitivity of tree species to drought. A low S (i.e. a steep slope) indicates that conduits rapidly cavitate in a narrow range of xylem tensions, while a high S (i.e. a shallow slope) indicates that conduits are more insensitive to drought (Fig. S6).

The final model that included slope, diameter at breast height (DBH), xylem pressure sensitivity (S) of the focal tree, the CWM_S_-DBH interaction and the CWM_S_-S interaction explained 41.6% of variance in TWD (Tables S3 and S4). Trees that grow on steep slopes suffer greater TWD. TWD of large trees decreased faster than that of small trees with increasing CWM_S_ (Fig. 1A). In addition, TWD of focal species with high S values increased, whereas those with low S values decreased, with increasing CWM_S_ (Fig. 1A), implying an effect of functional dissimilarity on TWD. We further directly tested the effect of functional dissimilarity and found that TWD of focal trees decreased as the distance of S (δTrait_s_) between neighbouring and focal trees increased (Fig. 1B).

**Figure 1.**
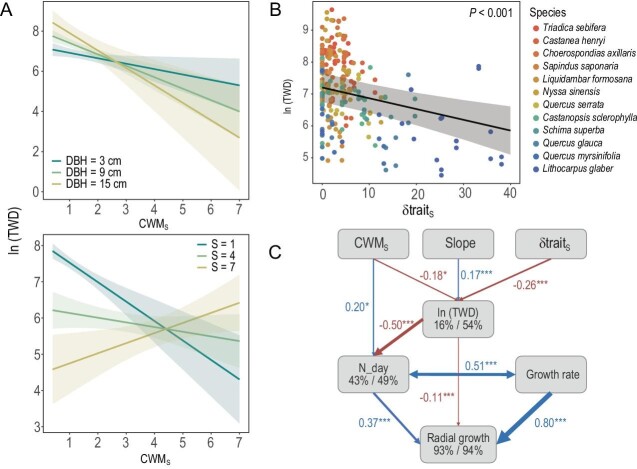
Neighbourhood effects on tree water deficit (TWD) and stem radial growth. TWD is natural-log transformed. (A) TWD as a function of the community-weighted mean of the xylem pressure sensitivity (S) of all trees in the local neighbourhood (CWM_S_). TWD of focal trees are fixed by different levels of DBH and values of S along CWM_S_; 95% confidence intervals are shown by shading. (B) Variation of focal tree's TWD with distance of xylem pressure sensitivity (δTrait_S_) between focal tree and neighbouring trees. Lines represent the fit of linear mixed-effects models; shading represents 95% confidence intervals. Species treated as random factors are shown in different colors. (C) Structural equation models (Fisher's C = 12.32, df = 12, *p* = 0.42) illustrating the influences of slope, community weighted mean (CWM_S_) and distances (δTrait_S_) of xylem pressure sensitivity (S) at the neighbourhood scale, TWD, number of days with stem growth (*N*_day) and growth rate during the growing days on the radial growths. Blue and red represent positive and negative effects, respectively. Significant standardized path-coefficients were calculated (***p < 0.001, **p < 0.01, *p < 0.05). Path-width was scaled by coefficient size. Marginal *R*^2^ (left) and conditional *R*^2^ (right) are shown below the response variables.

The results partially support our first hypothesis (H1) regarding the importance of functional dominance (measured as the CWM), but not functional diversity, influencing TWD of focal trees. With decreasing CWM_S_, the presence of more drought-sensitive trees within the neighbourhood increases the water deficit of focal trees because they may adopt more acquisitive water use

strategies, actively transpiring water for as long as it is supplied [11]. In addition, the effect of CWM on TWD depends on the size and xylem pressure sensitivity of the focal tree. The size dependence of neighbourhood effects likely occurred because larger trees typically have broader and deeper roots, providing a more stable water supply when water is limited, allowing them advantages during size-asymmetric water competition [6]. The trait distance of xylem pressure sensitivity between neighbouring and focal trees mitigates the water limitation of focal trees, consistent with the idea of the importance of hydrological niche segregation [12]. A mix of trees with different hydrological traits reduces niche overlap and soil water competition and may enhance facilitation among neighbouring trees. In addition, the functional dissimilarity of xylem pressure sensitivity is also correlated with dissimilarity in other key traits, such as whether a species is evergreen or deciduous (Fig. S7). As a result, phenological differences can create temporal complementarity for water use resulting from seasonal patterns during leaf expansion of deciduous species in spring and transpiration of evergreen trees in winter, as well as diurnal variation such as stomatal control of transpiration [13].

The result of the structural equation model (Fig. 1C) is consistent with our second hypothesis (H2), that neighbourhood effects regulate stem radial growth through TWD. TWD was positively correlated with slope (standardized path coefficient 0.17) and negatively correlated with both the dominance (CWM_S_) and trait distances (δTrait_S_) of xylem pressure sensitivity (standardized path coefficient −0.18 and −0.26, respectively). Radial growth increased with the number of days, with stem growth and growth rate increasing during the growing days. TWD had a direct negative effect on radial growth (standardized path coefficient −0.11) and an indirect negative effect via decreasing number of growing days (standardized path coefficient −0.50).

We found that TWD is a limiting factor for stem radial expansion. Physiological drought occurs not only during the dry season in subtropical forests, but can also arise under increased demands for transpiration due to high temperatures in summer [9]. Reduced water deficit due to neighbourhood effects and flat terrain provides more sufficient temporal water supply that is beneficial to tree radial expansion. In particular, TWD can limit radial growth by reducing the number of days with stem growth that largely determines annual growth [14]. Reduced TWD provides adequate turgor pressure for cell growth so that xylem cells do not get ‘stuck’ in the enlargement phase [15], and eventually achieves greater annual stem growth by accumulating the advantage in fine temporal resolution.

In summary, we investigated individual-mediated neighbourhood effects on TWD and the relationship between TWD and stem radial growth. The xylem pressure sensitivity, a trait that has received little attention in previous studies but contains important information regarding drought sensitivity, is closely associated with TWD. Collectively, the community weighted mean value of xylem pressure sensitivity at the neighbourhood scale, as well as difference of xylem pressure sensitivity between neighbouring and focal trees, plays key roles in regulating TWD. The reduction in TWD can promote radial growth by extending the window of time when trees grow. This highlights the positive effects of functional dissimilarity among individuals at the neighbourhood scale. As a result, a combination of more and less drought-sensitive species can benefit from growing together. These findings also have important implications for forestry, as they indicate that it might be useful to combine valuable-timber focal species with species that have functionally divergent hydraulic strategies. However, intraspecific trait variation and other characteristics such as canopy structure and belowground processes at the whole tree scale should be considered in further studies in order to deepen our understanding of resource complementarily mechanisms.

## Supplementary Material

nwad320_Supplemental_Files

## Data Availability

Data used in this study are available in the Supporting Information.
