# Supplementary material for: Functional dissimilarity in mixed forests promotes stem radial growth by mitigating tree water deficit
Source: Natl Sci Rev. 2023 Dec 18;11(3):nwad320. doi: 10.1093/nsr/nwad320 (PMC10936551; doi:10.1093/nsr/nwad320)
Supplement: nwad320_Supplemental_Files [file nwad320_supplemental_files.zip › Supplementary data.docx]

**Supplementary data for the manuscript “Functional dissimilarity in mixed forests promotes stem radial growth by mitigating tree water deficit”**

Hong-Tu Zhang(张宏图)^1^, Gheyur Gheyret(艾尤尔.亥热提)^1,2*^, Yun-Hao Bai(白云昊)^1^, Yanpei Guo(郭焱培)^1^, Shan Li(李珊)^3^, Bernhard Schmid^1,4^, Helge Bruelheide^5,6^, Keping Ma(马克平)^3^, Zhiyao Tang(唐志尧)^1*^

^1^ *Institute of Ecology, College of Urban and Environmental Sciences and Key Laboratory for Earth Surface Processes of Ministry of Education, Peking University, Beijing 100871, China*

^2^ *College of Geography and Tourism, Xinjiang Normal University, Urumqi 830054, China*

^3^ *State Key Laboratory of Vegetation and Environmental Change, Institute of Botany, Chinese Academy of Sciences, No. 20 Nanxincun, Xiangshan, Beijing 100093, China*

^4^ *Department of Geography, Remote Sensing Laboratories, University of Zurich, Winterthurerstrasse 190, 8057 Zurich, Switzerland*

^5^ *Institute of Biology/Geobotany and Botanical Garden, Martin Luther University Halle-Wittenberg, Halle, Germany*

^6^ *German Centre for Integrative Biodiversity Research (iDiv) Halle-Jena-Leipzig, Leipzig, Germany*

*Corresponding author.

*E-mail address:* [zytang@urban.pku.edu.cn](mailto:zytang@urban.pku.edu.cn) or [gheyur@pku.edu.cn](mailto:gheyur@pku.edu.cn).

**Supplementary materials and methods**

**Study site and radial growth measurements**

We conducted this study in the main part of the BEF-China experiment [1], which is a tree diversity manipulation platform that contains 0.067 ha plots planted and experimentally maintained with species richness treatments of 1, 2, 4, 8, 16, or 24 species. The BEF-China experiment is located in Xingangshan (29°08′–29°11′N, 117°90′–117°93′E), in the Jiangxi Province in southeast China. The region is characterized by a typical subtropical monsoon climate. The study was carried out at the experimental site A, which was established in 2009 and contained 261 plots with a size of 25.8 m × 25.8 m. Individuals of each species were randomly assigned to planting positions within a plot with between-tree distance of 1.29 m in horizontal projection (i.e., 400 individuals were initially planted in each plot). For more detailed information on the BEF-China experimental platform, see Bruelheide et al. [1] and Huang et al. [2]

To monitor radial growth of healthy trees, we installed automatic dendrometers (Ecomatik, Munich, Germany) on 293 individual trees of 15 focal species (nine deciduous and six evergreen species) at the end of December 2017. This included 2-4 individuals of each species in monoculture and in plots with different species richness levels. Dendrometers were installed at breast height (1.3 m) on focal trees after dead bark was removed to ensure tight contact between the sensor and the trunk. The accuracy of the dendrometer is 1.5~3.3 μm, and daily measurements were registered automatically every 30 min and saved in HOBO data loggers (UX120-006M, Onset Cooperation, USA). Dendrometer data was collected continuously through year 2018 and 2019. Firstly, we excluded outliers in raw dendrometer data with the R package “treenetproc” [3]. Secondly, we excluded incomplete data (more than 10% missing value for a whole year) and trees which had at least one neighbouring tree die (mortality rate in all neighbouring trees was 8.7%). Thirdly, three species, *Koelreuteria bipinnata*, *Castanopsis eyrei* and *Quercus serrata*, were excluded because of too fewer individuals in mixed-plant plots after the first step. Finally, a total of 160 focal trees of 12 species (seven deciduous and five evergreen species) were included in analysis (Table S1), as well as additional 11 species that were present as neighbouring species. For the remaining trees, we filled missing values with the R package ‘dendrometeR’ [4].

**Tree water deficit and growth characteristics**

High-temporal resolution automated band dendrometers are able to monitor diurnal changes of stem radius, and thus, allows the immediate assessment of the plant water responses. The diurnal changes in tree stem radius can be divided into irreversible processes (cell division and enlargement) and reversible processes (shrinkage and swelling induced by water absorption and loss in cells) [5]. We used the zero-growth approach [6] to separate tree water deficit (TWD) and radial growth (Figure S1). Zero-growth approach assumes the absence of growth during periods of stem shrinkage due to low turgor pressure. TWD is the difference between the maximum precedent stem radius and the actual stem radius, which provides information about plant water status and instant response to soil water [7]. We estimated growth as the increase in stem radius from its previous maximum. The start and end of the growing season were defined as the day of year when the cumulative stem radius values account for 5% and 95% of the total annual growth, respectively [8]. We also calculated two growth characteristics, the number of days with stem growth greater than zero within the growing season, as well as the mean daily growth rate during these days, which have important effects on tree radial growth [9].

**Plant functional traits**

We extracted 32 species-level functional traits from previous work at the same study site [10-12]. These traits included those related to the leaf economics spectrum(e.g., specific leaf area, leaf nitrogen content), stomata (e.g., conductance, size), mechanical properties (e.g., leaf toughness, wood density), and xylem hydraulics (e.g., water potential at which 50% loss of conductivity occurs) (Table S2). Leaf traits were measured on sun-exposed, fully developed, and non-damaged leaves from at least five individuals per species. Stomata traits were recorded by daily measurement on the same leaf from the same individual during dry spells in the summer and only on sunny days. Xylem hydraulic traits were measured in a pressure chamber using twigs from three individuals per species, and wood traits were measured from the same samples. In total, we used traits from 23 tree species in this analysis, including the 12 focal species, as well as additional 11 species that were present as neighbouring species. Because trait data for *Castanopsis carlesii* were missing, we used the average of trait data of species from the same genus, *Castanopsis sclerophylla* and *Castanopsis eyrei*, to fill the gap.

We calculated community-weighted mean (CWM) and functional diversity (FD) at the neighbourhood scale for each trait, using the function *dbFD* in the ‘FD’ package [13]. Neighbourhoods scale include the focal tree and neighbouring tree which is defined as the closest trees surrounding a focal tree with a maximum of eight neighbours [14].

$CWM=\sum_{i=1}^{n} P_{i}X_{i}$ (*Eq.1*)

$\mathrm{FD}=\sum_{i,j}^{n} d_{ij}P_{i}P_{j}$ (*Eq.2*)

where n is the number of species in the neighbourhood scale, P_i_ is proportion of species i in the neighbourhood scale, X_i_ is the trait value of species i, and d_ij_ is the Euclidean distance of trait dissimilarity between species i and j.

**Statistical analysis**

We included microtopography (slope, altitude and northness) of individuals that may have direct influence on soil moisture. High degree northness indicates that the slope is exposed to northern direction and receives less direct sunlight in the northern hemisphere [15]. The log-transformed annual TWD was fitted by microtopography, the size (DBH) and trait value (Trait_f_) of the focal tree, CWM and FD at the neighbourhood scale, and their interaction as follows:

$\ln\left( \mathrm{TWD} \right)\sim Slope+\mathrm{Altitude}+Northness+DBH+\mathrm{Trait}_{f}+CWM+FD+CWM:DBH+FD:DBH+CWM:\mathrm{Trait}_{f}+FD:\mathrm{Trait}_{f}$ (*Eq.3*)

We used each trait to fit the *Eq.3*, and then selected trait that explained the most variance in TWD (Table S2). We included the xylem pressure sensitivity (S), a hydraulic trait represented by the inverse slope of xylem vulnerability curve, in the full model (*Eq.3*) and fit models with all possible combinations of factors and then selected the model with the lowest AICc (Akaike Information Criterion corrected for small sample size) (Table S3) as the finial model (*Eq.4*). Type III sums of squares of the finial model were calculated given the significant interaction terms.

$\ln\left( \mathrm{TWD} \right)\sim Slope+DBH+S+\mathrm{CWM}_{S}+\mathrm{CWM}_{S}:DBH+\mathrm{CWM}_{S}:S$ (*Eq. 4*)

Because we found a significant interaction CWM_S_-S, indicating effect of functional dissimilarity between focal tree and neighbouring trees. Therefore, we calculated the trait distance of xylem pressure sensitivity (S) (δTrait_S_) between the focal tree (S_f_) and eight neighbouring trees (S_n_) [16] as:

$\mathrm{Trai}t_{S}=\sum_{n=1}^{8} \left| S_{f}-S_{n} \right|$ (*Eq.5*)

We then used a linear mixed-effects model to fit TWD with the function *lmer* in the ‘lme4’ package [17], with δTrait_S_ as fixed term and species as random factor:

$\ln\left( \mathrm{TWD} \right)\sim\mathrm{Trai}t_{S}+\left( 1 | \mathrm{Species} \right)$ *(Eq.6)*

We developed a structural equation model (SEM) with species as the random intercept using the ‘piecesewiseSEM’ package [18]. Based on previous analysis, we included the slope and the CWM (CWM_S_) and trait distance (δTrait_S_) of xylem pressure sensitivity (S) into the SEM (Figure S2). We examined the influence of the number of days with growth within the growing season and mean daily growth rate during the growing days [9]. We fitted the global model via Fisher’s C statistic (P>0.05) and calculated the standardized path coefficients of each path. Non-significant paths (P>0.05) were removed in finial SEM model and significant paths of the missing relationships were included by using the tests of directed separation to evaluate whether the path coefficients are significantly different from zero.

**Table S1.** Basic information of the focal trees. Species-species characteristics of annual tree water deficit and stem radial growth (median with 25%–75% interquartile range in brackets). NR: neighbourhood species richness of trees; TWD: tree water deficit accumulation within a year; N_day: number of days with stem growth within the growing season; Daily growth rate: mean daily radial growth during N_day; DBH: diameter at breast height.

| Species | Life habit | No. of Trees | No. of Records | NR | TWD (mm) | N_day  (days) | Daily growth rate (mm/day) | Radial growth  (mm/yr) | DBH (cm) |
| --- | --- | --- | --- | --- | --- | --- | --- | --- | --- |
| *Triadica sebifera* | D | 11 | 21 | 2,3,4,6,7,8 | 3969.40  (2976.81-5353.91) | 27  (15-71) | 0.020  (0.017-0.037) | 0.40  (0.25-2.18) | 5.8  (4.7-7.1) |
| *Castanea henryi* | D | 16 | 27 | 1,2,4,5,6 | 1879.03  (1267.22-3001.37) | 118  (66-148) | 0.043  (0.027-0.073) | 5.06  (1.75-10.36) | 6.5  (4.2-8.8) |
| *Choerospondias axillaris* | D | 20 | 31 | 1,2,3,4,5,6,7 | 3836.25  (2868.51-6404.5) | 69  (34-88) | 0.049  (0.031-0.07) | 2.83  (1.59-6.09) | 9.3  (7.6-10.5) |
| *Sapindus saponaria* | D | 14 | 25 | 1,2,4,5,6,7,8 | 1569.90  (1140.26-2121.31) | 71  (48-88) | 0.026  (0.020-0.047) | 2.05  (1.25-4.48) | 5.7  (4.6-5.9) |
| *Liquidambar formosana* | D | 17 | 25 | 1,2,3,4,5,6,7 | 918.26  (430.79-2141.83) | 130  (106-142) | 0.038  (0.029-0.046) | 5.07  (3.43-6.74) | 5.8  (4.7-7.0) |
| *Nyssa sinensis* | D | 14 | 24 | 1,2,3,4,5,6,7 | 1778.17  (963.86-4679.28) | 90  (58-131) | 0.043  (0.030-0.054) | 3.79  (2.23-6.08) | 7.8  (6.3-8.5) |
| *Quercus serrata* | D | 11 | 18 | 1,2,3,4,6,7 | 1141.83  (655.32-1487.58) | 122  (76-153) | 0.020  (0.019-0.034) | 2.92  (2.09-3.88) | 4.7  (3.5-5.4) |
| *Castanopsis sclerophylla* | E | 15 | 23 | 1,2,3,4,6,8 | 978.11  (670.16-1294.57) | 138  (108-163) | 0.046  (0.023-0.059) | 5.28  (3.26-8.33) | 4.4  (3.5-5.6) |
| *Schima superba* | E | 10 | 14 | 1,2,5,6 | 815.04  (562.83-967.88) | 148  (121-186) | 0.025  (0.019-0.042) | 3.77  (2.38-7.43) | 6.8  (6.1-7.5) |
| *Quercus glauca* | E | 10 | 13 | 1,2,4,5 | 300.02  (208.34-456.62) | 198  (162-215) | 0.026  (0.022-0.03) | 5.25  (4.48-5.74) | 4.9  (4.1-5.7) |
| *Quercus myrsinifolia* | E | 8 | 10 | 1,2,5 | 345.84  (185.61-560.57) | 188  (159-190) | 0.032  (0.028-0.043) | 5.53  (4.18-8.37) | 6.0  (3.9-7.2) |
| *Lithocarpus glaber* | E | 14 | 19 | 1,2,3,5,6,7 | 267.51  (148.31-471.53) | 201  (174-218) | 0.029  (0.025-0.035) | 6.30  (4.70-8.03) | 5.5  (4.0-6.3) |

D=deciduous, E=evergreen

**Table S2.** The performance of the full model (*Eq.3*) with different traits. Trait data was extracted from Kröber et al. [7] AICc: Akaike Information Criterion corrected for small sample size.

| Trait | Abbr. | R^2^ | AICc |
| --- | --- | --- | --- |
| Xylem pressure sensitivity (the inverse slope of xylem vulnerability curve) | S | 0.438 | 646.38 |
| Leaf carbon nitrogen ratio | CN | 0.437 | 646.98 |
| Leaf nitrogen content | LNC | 0.414 | 656.71 |
| Leaf toughness | LEAFT | 0.408 | 659.53 |
| Leaf habit |  | 0.400 | 662.68 |
| Leaf magnesium content | MG | 0.385 | 668.93 |
| Absolute fitted Max. stomatal conductance | CONMAXFITA | 0.379 | 671.35 |
| Spongy parenchyma thickness | SPONGY | 0.356 | 680.63 |
| Leaf carbon content | LCC | 0.333 | 689.28 |
| Leaf thickness | LEAFTHICK | 0.330 | 690.20 |
| Water potential at which 50% loss of conductivity occurs | Ψ_50_ | 0.320 | 694.11 |
| Upper epidermis thickness | UPPEREPI | 0.319 | 694.43 |
| Average stomatal conductance | CONMEAN | 0.303 | 700.34 |
| Specific leaf area | SLA | 0.299 | 701.60 |
| Leaf calcium content | CA | 0.297 | 702.36 |
| Maximum flowrate | K_S­_ | 0.293 | 703.81 |
| Relative fitted Max. stomatal conductance | CONMAXFIT | 0.272 | 711.23 |
| Leaf area | LA | 0.271 | 711.37 |
| Leaf dry matter content | LDMC | 0.264 | 713.81 |
| Maximum stomatal conductance | CONMAX | 0.262 | 714.48 |
| Vpd at CONMAXFIT | VPDMAXFIT | 0.248 | 719.15 |
| Length of first-order veins per cm^2^ | VEINLENGTH | 0.245 | 720.21 |
| Leaf potassium content | K | 0.234 | 723.92 |
| Vpd at point of inflection of fitted stomatal conductance | VPDPOI | 0.234 | 723.94 |
| Log ratio of the palisade to spongy parenchyma thickness | LOG10RATIO | 0.228 | 725.81 |
| Vpd at CONMAX | VPDMAX | 0.223 | 727.28 |
| Stomata size, ellipse from stomata length and width | STOMSIZE | 0.220 | 728.49 |
| Wood density | WOODDENS | 0.205 | 733.12 |
| Water potential | WPOT | 0.202 | 734.06 |
| Stomata index | STOIND | 0.185 | 739.31 |
| Palisade parenchyma thickness | PALIS | 0.176 | 741.96 |
| Stomata density | STOMDENS | 0.150 | 750.00 |

**Table S3.** Models with all possible combinations of factors from the full model (*Eq.3*) were fitted and ranked based on Akaike Information Criterion corrected for small sample size (AICc). Top ten sub models with minimal AICc are shown. The signal indicates that the variable is included in sub model. DBH: diameter at breast height of the focal tree; S: xylem pressure sensitivity of the focal tree; CWM_S_: community-weighted mean of S at the neighbourhood scale; FD_S_: functional diversity of S at the neighbourhood scale.

| Slope | Altitude | Northness | DBH | S | CWM_S_ | FD_S_ | CWM_S_:DBH | FD_S_:DBH | CWM_S_: S | FD_S_:S | logLik | ΔAICc |
| --- | --- | --- | --- | --- | --- | --- | --- | --- | --- | --- | --- | --- |
| × |  |  | × | × | × |  | × |  | × |  | -311.13 | 0.00 |
| × |  | × | × | × | × |  | × |  | × |  | -310.74 | 1.39 |
| × | × |  | × | × | × |  | × |  | × |  | -311.11 | 2.13 |
| × |  |  | × | × | × | × | × |  | × |  | -311.13 | 2.15 |
| × |  |  | × | × | × |  |  |  | × |  | -313.41 | 2.43 |
| × |  |  | × | × | × | × | × |  | × | × | -310.32 | 2.70 |
| × |  |  | × | × | × | × |  | × | × |  | -311.54 | 2.98 |
| × |  |  | × | × | × | × |  | × | × | × | -311.55 | 3.17 |
| × | × | × | × | × | × |  | × |  | × |  | -310.71 | 3.49 |
| × |  | × | × | × | × | × | × |  | × |  | -310.74 | 3.56 |

**Table S4.** Type III ANOVA parameters and regression coefficients for the best-fitting model (R^2^=41.6%). S: xylem pressure sensitivity (represented by the inverse slope of xylem vulnerability curve) of the focal tree; DBH: diameter at breast height of the focal tree; CWM_S_: community-weighted mean of S at the neighbourhood scale.

| Parameter | SS | df | F value | p (>F) | Estimate |
| --- | --- | --- | --- | --- | --- |
| Intercept | 284.66 | 1 | 392.19 | **<0.001** | 7.27 |
| Slope | 5.44 | 1 | 7.49 | **0.007** | 0.02 |
| DBH | 7.89 | 1 | 10.87 | **0.001** | 0.13 |
| S | 35.72 | 1 | 49.22 | **<0.001** | -0.60 |
| CWM_S_ | 2.58 | 1 | 3.55 | 0.06 | -0.35 |
| CWM_S_×DBH | 3.25 | 1 | 4.47 | **0.04** | -0.05 |
| CWM_S_×S | 22.56 | 1 | 33.83 | **<0.001** | 0.14 |

**
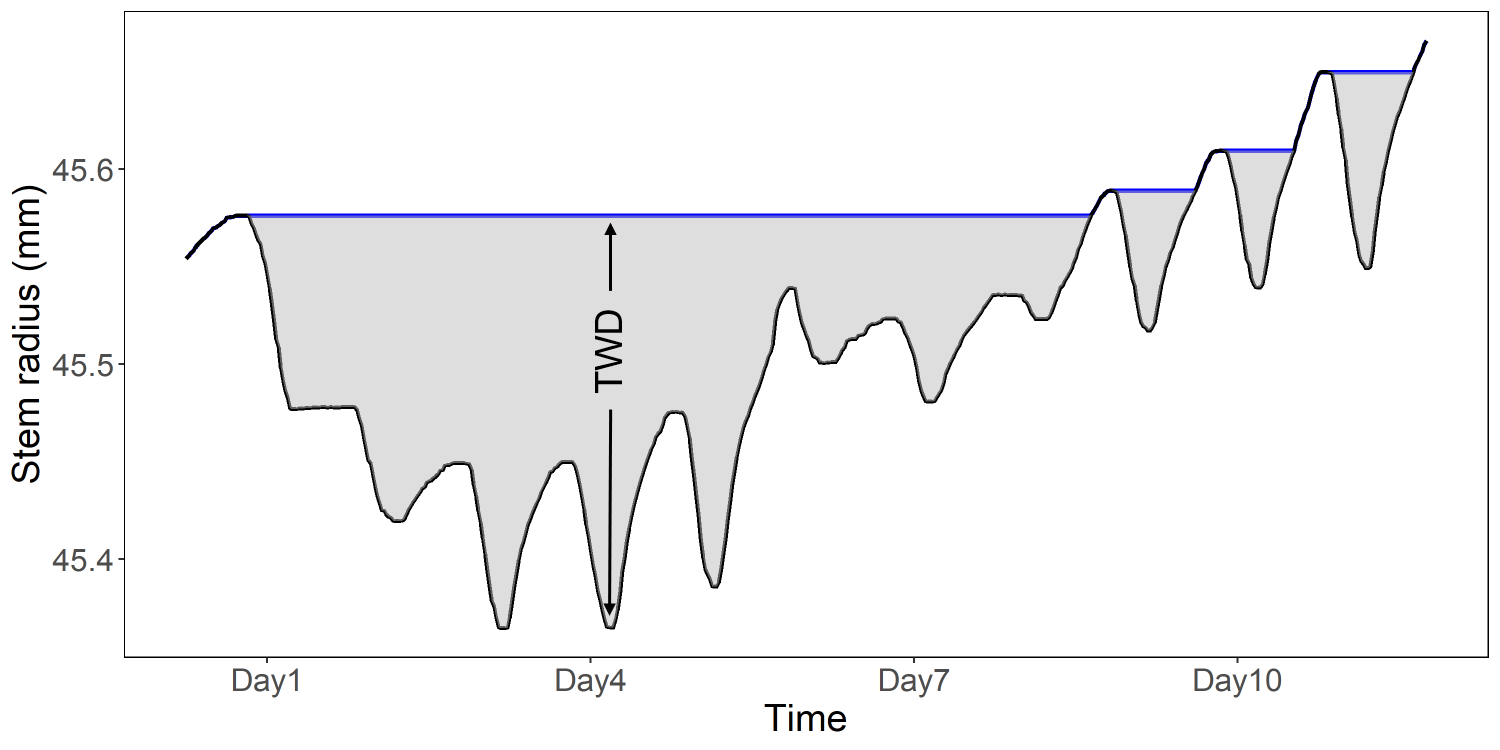
**

**Figure S1.** Stem radius variation of one of our trees over several days. Separate tree water deficit (TWD) and stem radial growth through zero-growth (ZG) approach. Black line indicates stem radius; blue line indicates radial growth; the shadow area between black line and blue line indicates TWD.


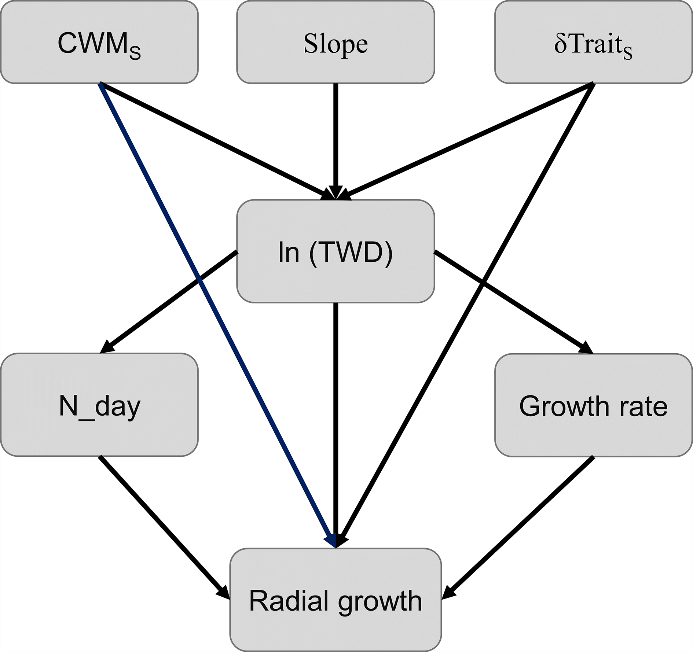


**Figure S2.** Hypotheses framework showing direct and indirect effects on tree radial growth. Directional arrows represent hypothesized pathways. Slope, community weighted mean (CWM_S_) of the neighbors and the distances (δTrait_S_) of xylem pressure vulnerability (S, the inverse slope of xylem vulnerability curve) between neighbors and the focal trees influence tree water deficit (TWD). Tree water deficit directly influence stem radial growth and has indirect effects on stem radial growth through tree growth characteristic: the number of days with stem growth (N_day) and mean daily growth rate during the growing days.

**
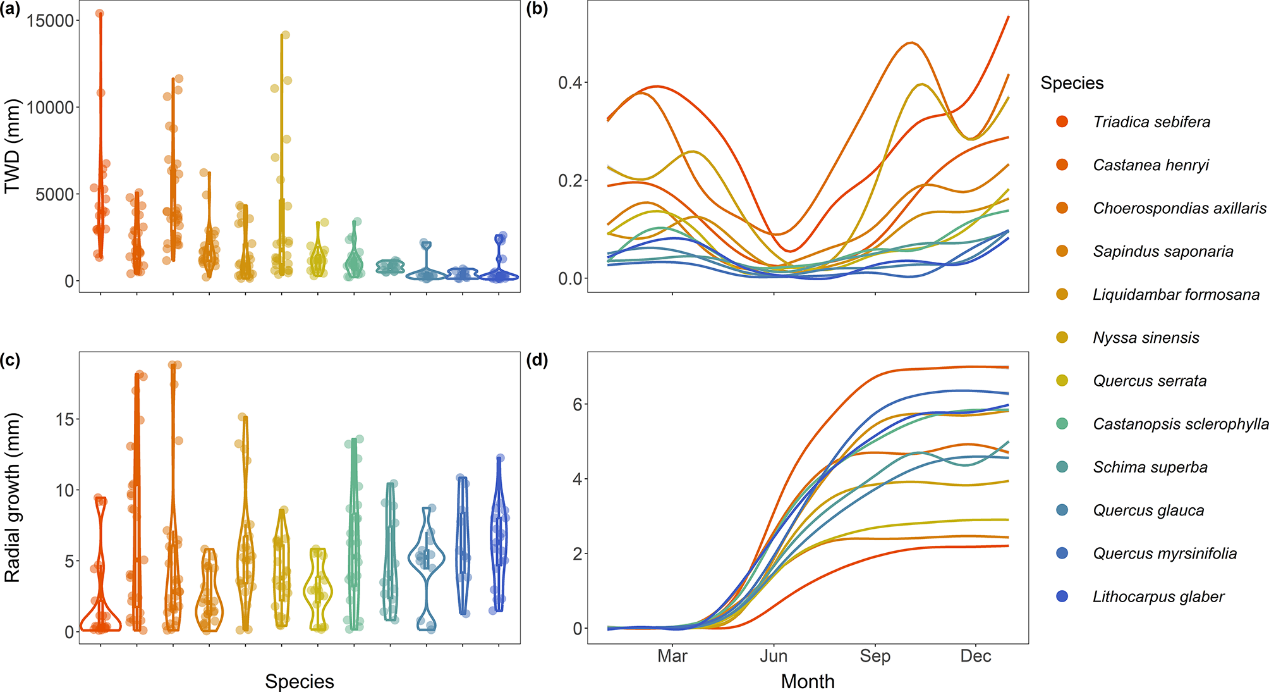
**

**Figure S3.** (a) Annual TWD and (c) annual radial growth for species. (b) TWD variation and (d) radial growth accumulation within a year. TWD and radial growth accumulation were fitted with general generalized additive model. Species are represented from red to yellow for deciduous species and from green to blue for evergreen species.

**
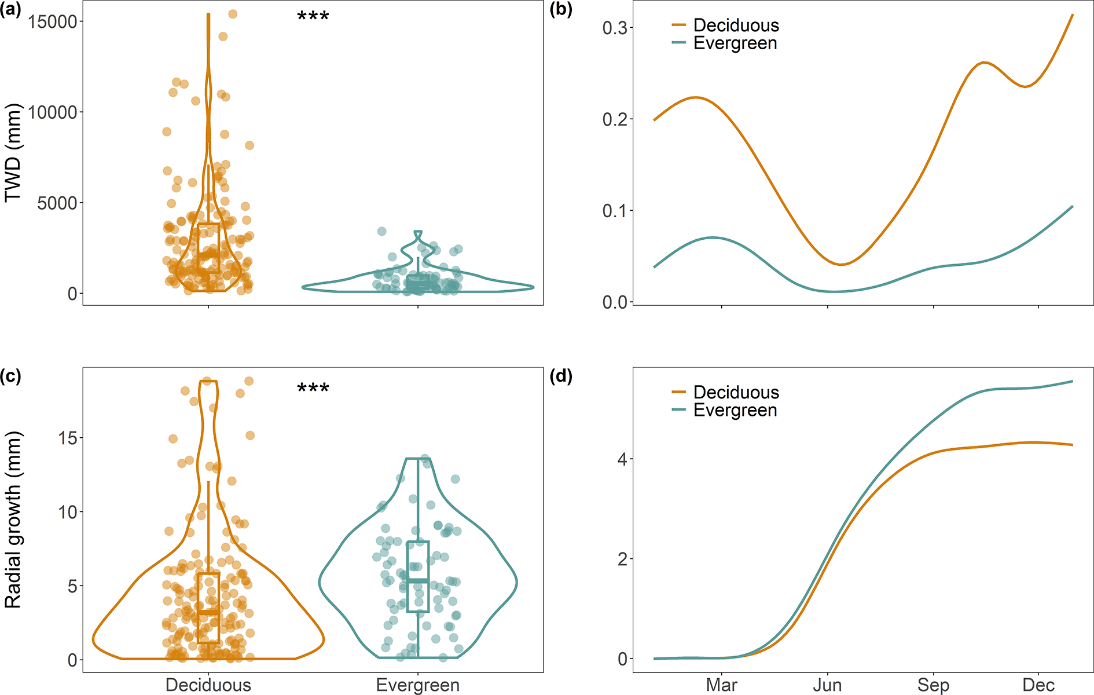
**

**Figure S4.** Annual and variation within a year of TWD (a & b) and radial growth (c & d) for evergreen and deciduous species. Difference in annual TWD and radial growth of evergreen and deciduous species compared by Wilcoxon test. TWD and radial growth accumulation were fitted with general generalized additive model.

**
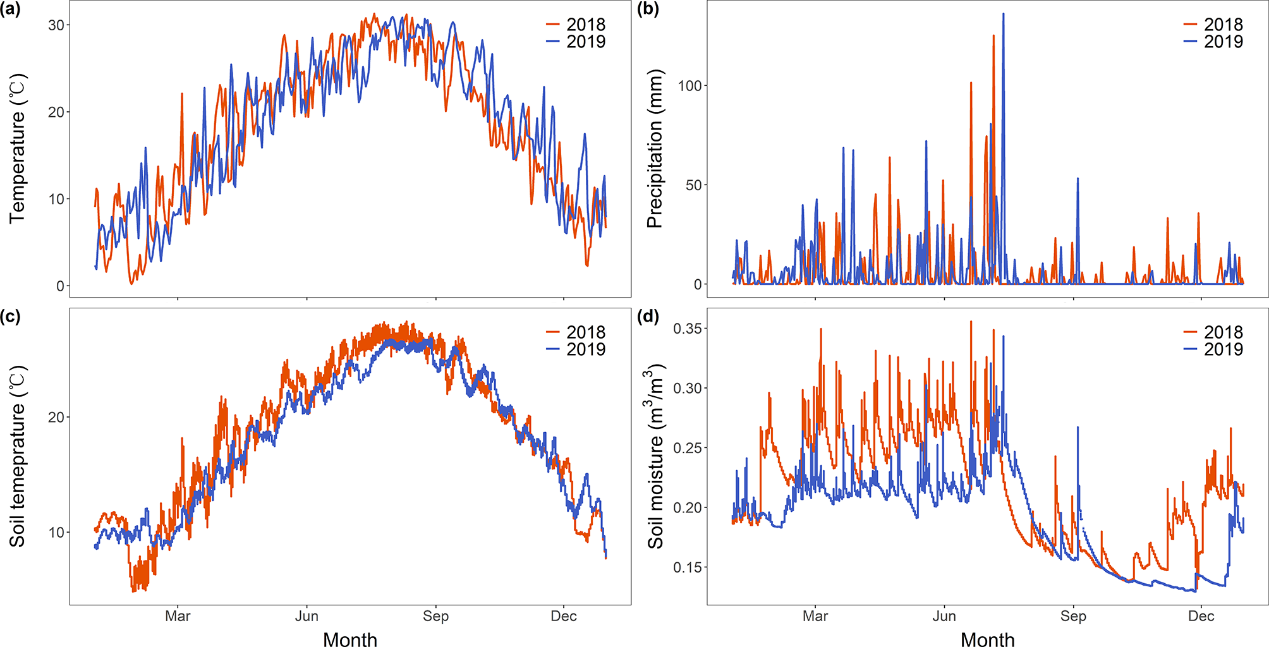
**

**Figure S5.** Environment conditions during year 2018 and 2019. (a) Daily mean temperature and (b) daily precipitation from Dexing Station (28°57′ N, 117°35′ E), the nearest national standard meteorological station to BEF-China. (c) Soil temperature and (d) soil moisture at half hour intervals from EM50 devices. EM50 were installed at nine plots of our studied trees within BEF-China. The soil data was averaged to provide an overall picture of the soil conditions.

**
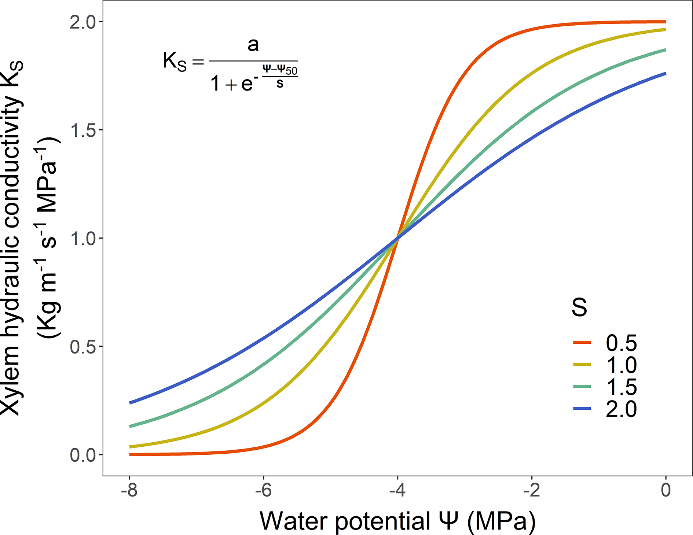
**

**Figure S6.** An illustration of the variation of xylem vulnerability curves with different xylem pressure sensitivity (S). Xylem vulnerability curves show variation of the loss of specific xylem hydraulic conductivity (K_S_) as a function of water potential (Ψ). S is defined as the inverse slope of xylem vulnerability curve fitted with sigmoid regression and indicates the xylem pressure sensitivity [6]. Parameter a (original maximum specific xylem hydraulic conductivity) and Ψ_50_ (water potential at which 50% loss of conductivity occurs) are fixed (a=2 Kg m^-1^ s^-1^ MPa^-1^, Ψ_50_=-4 MPa).

**
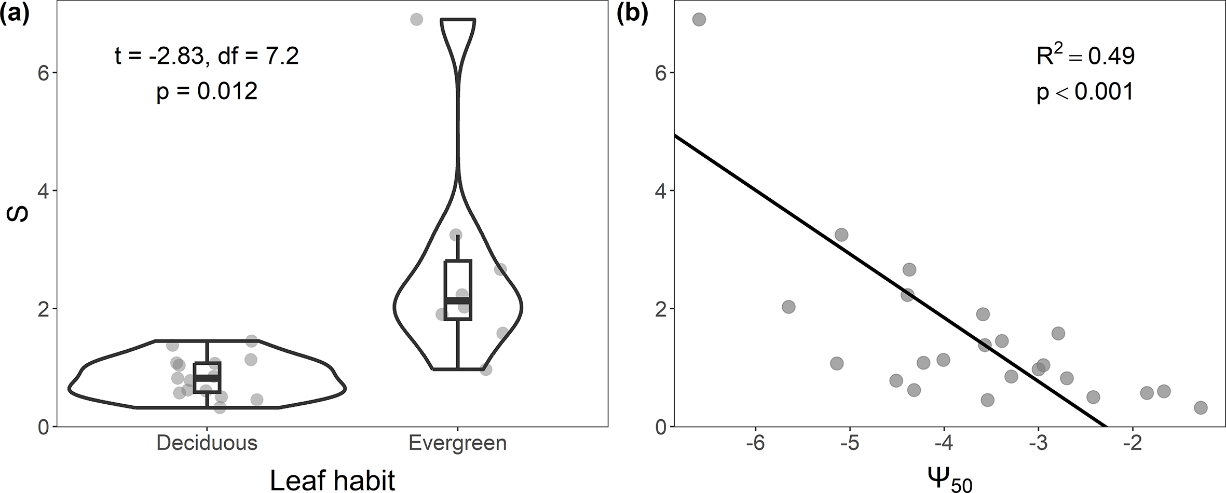
**

**Figure S7** (a) Differences of xylem pressure sensitivity (S) between different leaf habits and (b) its relationship with Ψ_50_ (water potential at which 50% loss of conductivity occurs).

**References**

1. Bruelheide H, Nadrowski K, and Assmann T et al. Designing forest biodiversity experiments: general considerations illustrated by a new large experiment in subtropical China. *Methods Ecol Evol* 2014; **5**: 74-89.
2. Huang Y, Chen Y, and Castro-Izaguirre N et al. Impacts of species richness on productivity in a large-scale subtropical forest experiment. *Science* 2018; **362**: 80-83.
3. Knüsel S, Peters RL, and Haeni M. et al. Processing and extraction of seasonal tree physiological parameters from stem radius time series. *Forests* 2021; **12:** 765.
4. van der Maaten E, van der Maaten-Theunissen M, and Smiljanic M et al. dendrometeR: Analyzing the pulse of trees in R. *Dendrochronologia* 2016; **40:** 12-16.
5. Zweifel R, Zimmermann L, and Newbery DM. Modeling tree water deficit from microclimate: an approach to quantifying drought stress. *Tree Physiol* 2005; **25:** 147-156.
6. Zweifel R, Haeni M, and Buchmann N et al. Are trees able to grow in periods of stem shrinkage? *New Phytol* 2016; **211:** 839-849.
7. Nehemy MF, Benettin P, and Asadollahi M et al. Tree water deficit and dynamic source water partitioning. *Hydro Processes* 2021; **35**: e14004.
8. Knüsel S, Peters RL, and Haeni M et al. Processing and extraction of seasonal tree physiological parameters from stem radius time series. *Forests* 2021; **12:** 765.
9. Etzold S, Sterck F, and Bose AK et al. Number of growth days and not length of the growth period determines radial stem growth of temperate trees. *Ecol Lett* 2021; **25**: 427-439.
10. Kröber W, and Bruelheide H. Transpiration and stomatal control: a cross-species study of leaf traits in 39 evergreen and deciduous broadleaved subtropical tree species. *Trees* 2014; **28:** 901-914.
11. Kröber W, Zhang S, and Ehmig M et al. Linking xylem hydraulic conductivity and vulnerability to the leaf economics spectrum-a cross-species study of 39 evergreen and deciduous broadleaved subtropical tree species. *PLoS One* 2014; **9:** e109211
12. Kröber W, Li Y, and Hardtle W et al. Early subtropical forest growth is driven by community mean trait values and functional diversity rather than the abiotic environment. *Ecol Evol* 2015; **5:** 3541-3556.
13. Laliberté E, Legendre P, and Shipley B. Measuring functional diversity (FD) from multiple traits, and other tools for functional ecology. *R package version 1.0-12* 2014.
14. Fichtner A, Hardtle W, and Bruelheide H et al. Neighbourhood interactions drive overyielding in mixed-species tree communities. *Nat Commun* 2018; **9**: 1144.
15. Li Y, Härdtle W, and Bruelheide H et al. Site and neighborhood effects on growth of tree saplings in subtropical plantations (China). *For Ecol Manag* 2014; 327: 118-127.
16. Roscher C, Gubsch M, and Lipowsky A et al. Trait means, trait plasticity and trait differences to other species jointly explain species performances in grasslands of varying diversity. *Oikos* 2018; **127**: 855-865.
17. Bates D, Maechler M, and Bolker B et al. Fitting linear mixed-effects models using lme4. *J Stat Softw* 2015; **67**: 1-48.
18. Lefcheck JS. PIECEWISESEM: Piecewise structural equation modelling in R for ecology, evolution, and systematics. *Methods Ecol Evol* 2016; **7**: 573-579.
